# Nutritional Recovery Promotes Hypothalamic Inflammation in Rats during Adulthood

**DOI:** 10.1155/2014/736506

**Published:** 2014-08-31

**Authors:** Hellen Barbosa Farias Silva, Ana Paula Carli de Almeida, Katarine Barbosa Cardoso, Letícia Martins Ignacio-Souza, Silvia Regina de Lima Reis, Marise Auxiliadora de Barros Reis, Márcia Queiroz Latorraca, Marciane Milanski, Vanessa Cristina Arantes

**Affiliations:** ^1^Mestrado em Biociências, Faculdade de Nutrição, Universidade Federal de Mato Grosso, Cuiabá, MT, Brazil; ^2^Laboratório de Avaliação Biológica de Alimentos, Faculdade de Nutrição, Universidade Federal de Mato Grosso, Cuiabá, MT, Brazil; ^3^Departamento de Alimentos e Nutrição, Faculdade de Nutrição, Universidade Federal de Mato Grosso, Avenida Fernando Correa da Costa, 2367. Bairro Boa Esperança, Cuiabá, MT, Brazil; ^4^Faculdade de Ciências Aplicadas da Universidade Estadual de Campinas, Campinas, SP, Brazil

## Abstract

We evaluated whether protein restriction in fetal life alters food intake and glucose homeostasis in adulthood by interfering with insulin signal transduction through proinflammatory mechanisms in the hypothalamus and peripheral tissues. Rats were divided into the following: a control group (C); a recovered group (R); and a low protein (LP) group. Relative food intake was greater and serum leptin was diminished in LP and R compared to C rats. Proinflammatory genes and POMC mRNA were upregulated in the hypothalamus of R group. Hypothalamic NPY mRNA expression was greater but AKT phosphorylation was diminished in the LP than in the C rats. In muscle, AKT phosphorylation was higher in restricted than in control animals. The HOMA-IR was decreased in R and C compared to the LP group. In contrast, the *K*_itt_ in R was similar to that in C and both were lower than LP rats. Thus, nutritional recovery did not alter glucose homeostasis but produced middle hyperphagia, possibly due to increased anorexigenic neuropeptide expression that counteracted the hypothalamic inflammatory process. In long term protein deprived rats, hyperphagia most likely resulted from increased orexigenic neuropeptide expression, and glucose homeostasis was maintained, at least in part, at the expense of increased muscle insulin sensitivity.

## 1. Introduction

Epidemiological and animal studies support a relationship between poor fetal growth and the subsequent development of obesity, type 2 diabetes, and metabolic syndrome [1–4]. Such developmental programming has been explained by the “thrifty phenotype” hypothesis, which proposes that poor fetal nutrition can result in reprogramming of the fetus, which allows the offspring to maximize the body's capacity for energy storage under conditions of poor nutrition once out of the womb. However, this phenotype would be detrimental under conditions where normal or excessive nutrition are present and would thus promote obesity [2, 5].

Body weight, food intake, and metabolism are regulated by the hypothalamus, which processes central and peripheral signals. Within the hypothalamus, neurons residing in the ARC (arcuate nucleus), PVN (paraventricular), and PF/LH (perifornical/lateral hypothalamus) axis communicate with each other and are subject to the influence of several peripheral factors, including leptin and insulin [6]. The effect of these hormones on food intake occurs in part by convergence on a specific set of neurons within the ARC [7, 8] that contains neuronal populations expressing orexigenic neuropeptide Y (NPY) and agouti-related peptide (AgRP) and the anorectic proopiomelanocortin (POMC). Increased NPY synthesis and secretion and reduced expression of POMC and its cleaved product α-MSH [9] are characteristic of various models of obesity [10, 11]. These changes may be due to disturbances in the production and release of leptin and insulin. Inappropriate concentrations of or a shift in these hormones due to poor fetal nutrition during a critical window of neuronal development and feeding pathway differentiation may have permanent structural consequences [12]. Moreover, increased hypothalamic inflammation may also contribute to increased susceptibility to obesity in rats exposed to early malnutrition. It has been shown that a low protein diet during lactation interferes with the innate immune response in adulthood, imprinting permanent alterations on cytokine production. These animals exhibited high circulating levels of tumor necrosis factor (TNF)-α and an increased expression of TNF-α mRNA in the spleen and liver [13]. TNF-α induces hypothalamic and peripheral insulin resistance in rodents [14–16] and alters insulin sensitivity and glucose homeostasis in humans [17, 18]. In obese mice, the inhibition of hypothalamic inflammation by immunoneutralizing antibodies against TNFα and Toll-like receptor (TLR) 4 improved insulin signal transduction in the liver [16]. In previous studies [19], we verified that adult rats were exposed to a low protein diet during intrauterine life and lactation and then fed a regular diet after weaning exhibited elevated food intake but did not express the obesity phenotype or glucose intolerance. It has been proposed that the degree of mismatching between the pre- and postnatal environment is a major determinant of subsequent disease [20, 21] and that the lactation period is a critical time for increasing the risk of obesity and insulin resistance, even in obesity-resistant animals [22]. Thus, we evaluated whether protein restriction in fetal life alters food intake and glucose homeostasis in adulthood by interfering with insulin signal transduction in the hypothalamus and peripheral tissues through proinflammatory mechanisms.

## 2. Materials and Methods

### 2.1. Animals and Diets

All experimental procedures involving rats were performed in accordance with the guidelines of the Brazilian Society of Science in Animals of Laboratory (SBCAL) and were approved by the ethics committee at the Federal University of Mato Grosso (protocol No. 23108.051511/10-0). Male and virgin female Wistar rats (85–90 days old) were obtained from the university's breeding colony. Mating was accomplished by housing females with males overnight, and pregnancy was confirmed by examining vaginal smears for the presence of sperm. Pregnant females were separated at random and fed from day 1 of pregnancy until the end of lactation with an isoenergetic diet containing either 6% protein (low protein diet, LP) or 17% protein (control diet). The protein in the LP diet was replaced by the same amount of carbohydrate, as described previously [19]. Spontaneous delivery took place at day 22 of pregnancy, and at 3 days of age, large litters were reduced to eight pups each to ensure a standard litter size per mother. After birth, the males were divided into three groups: (1) a control group (C) consisting of rats born to and suckled by dams fed the control diet during pregnancy, lactation, and after weaning; (2) a recovered group (R) consisting of the offspring of dams fed an LP diet during pregnancy but fed the control diet during lactation and after weaning; and (3) an LP group consisting of the offspring of dams fed a LP diet during pregnancy and lactation and after weaning. During the experimental period, the rats were fed* ad libitum *with their respective diets and had free access to water. They were kept under standard lighting conditions (12 hours light: dark cycle) at 24°C. Food intake and body weight were recorded weekly, and the data are expressed as absolute and relative values. Absolute food intake refers to food consumed during ten weeks of the experimental period. To assess the relative food intake, the food intake was normalized per 100 g body weight at week ten of the experimental period. At the end of the experimental period, the rats were subjected to glucose and insulin tolerance test. After the tests, the rats were killed by decapitation, and their blood was collected to perform biochemical and hormonal analyses of the hypothalamus, liver, and muscle.

#### 2.1.1. Glucose Tolerance Test

After 12 hours of fasting, glucose (200 g/L) was administered intraperitoneally at a dose of 2 g/kg of body weight. Blood samples were obtained from an incision at the tip of the tail 0, 30, 60, 90, and 120 minutes after glucose administration to determine serum glucose concentrations (Accu-Chek portable glucose meter, Roche Diagnostics, Germany). The glucose response during the glucose tolerance test was calculated by estimating the total area under the glucose (Δ*G*) curve using the trapezoidal method [23].

#### 2.1.2. Insulin Tolerance Test

After 12 hours of fasting, insulin (regular) was administered intraperitoneally at a dose of 1.5 U/kg of body weight. Blood samples were obtained from a cut at the tip of the tail 0, 5, 10, and 15 minutes after insulin administration to determine serum glucose concentrations (Accu-Chek portable glucose meter,Roche Diagnostics, Germany). Glucose responses during the insulin tolerance test were evaluated by the constant of the disappearance of plasma glucose (*K*_itt_), which was calculated from the slope of the decrease in log-transformed plasma glucose between 0 and 15 minutes [24] after insulin administration, when the glucose concentration declined linearly.

### 2.2. Organ Weighs and Liver Glycogen Content

After medial laparotomy, epididymal white adipose and liver tissue were quickly removed and their fresh weight was determined. Liver aliquots were frozen immediately in liquid nitrogen and stored at −80°C to determine hepatic fat [25] and glycogen [26] contents.

### 2.3. Biochemical and Hormonal Profile

Blood samples were collected and centrifuged to 13.000 RPM for 30 minutes. Sera were stored at −80°C for the subsequent measurement of serum albumin concentrations by the colorimetric method [27] to characterize the nutritional status of the animals. Commercial ELISA kits were used to measure serum insulin (rat/mouse insulin—Cat.# EZRMI-13 K—Millipore) and leptin (rat leptin—Cat.# EZRL-83 K—Millipore) concentrations.

### 2.4. RNA Extraction, Real Time PCR and PCR Array

Hypothalamic total RNA was extracted using Trizol reagent (Life Technologies, Gaithersburg, MD, USA), according to the manufacturer's recommendations. The RNA integrity was checked by agarose gel electrophoresis. The synthesis of cDNA was conducted using 3 *μ*g of total RNA and the High-Capacity cDNA Reverse Transcription Kit (Applied Biosystems). Samples obtained from three hypothalami from C, R, and LP groups were analyzed using a real-time PCR array (Rat Inflammatory Cytokines Receptors RT² Profiler-SuperArray Bioscience Corp., Frederick, MD, USA) containing 84 genes related to this condition plus five housekeeping genes. Controls are also included on each array for genomic DNA contamination, RNA quality, and general PCR performance as shown in http://www.sabiosciences.com/rt_pcr_product/HTML/PARN-011A.html. To confirm the data obtained from PCR array, some genes were selected to perform an individual real-time PCR analysis. Thus, the optimal concentration of cDNA and primers and the maximum efficiency of amplification were obtained through five-point, twofold dilution curve analysis for each gene. Each PCR contained 20–50 ng of reverse-transcribed RNA and was run according to the manufacturer's recommendation using the TaqMan PCR Master Mix (Applied Biosystems). Intron-skipping primers were obtained from Applied Biosystems (TNFα Rn00562055_m1, NPY Rn00561681_m1, IL1*β* Rn00580432_m1, POMC Rn00595020_m1). Glyceraldehyde-3-phosphate dehydrogenase (GAPDH number 4352338E) primers were used as controls. No significant change in GAPDH expression was detected in the different experimental conditions. The real-time PCR analysis was conducted on ABI Prism 7500 detection system (Applied Biosystems). The data were analyzed using the system Sequence Detector 1.7 (Applied Biosystems).

### 2.5. Immunoblotting

For experiments that measured insulin signaling through AKT phosphorylation, the abdominal cavities of anesthetized rats were opened, and the animals received a bolus injection of saline (100 *μ*L) (-) or insulin (100 *μ*L, 10^−6^ M) intravenously in a cava vein. Liver, soleus muscle, and hypothalamus specimens were removed 45 seconds, 90 seconds, and 15 minutes, respectively, after the insulin injection and immediately homogenized by sonication (15 s) in a freshly prepared antiprotease cocktail (10 mmol/L imidazole, pH 8.0, 4 mmol/L EDTA, 1 mmol/L EGTA, 0.5 g/L pepstatin A, 2 g/L aprotinin, 2.5 mg/L leupeptin, 30 mg/L trypsin inhibitor, 200 *μ*mol/L DL-dithiothreitol, and 200 *μ*mol/L phenylmethylsulfonyl fluoride). After sonication, an aliquot of extract was collected, and the total protein content was determined by the dye-binding protein assay kit (Bio-Rad Laboratories, Hercules, CA). The samples were incubated for 5 minutes at 80°C with 4x concentrated Laemmli sample buffer (1 mmol sodium phosphate/L, pH 7.8, 0.1% bromophenol blue, 50% glycerol, 10% SDS, and 2% mercaptoethanol) and then run on 10% polyacrylamide gels at 120 V for 30 minutes. The electrotransfer of proteins to nitrocellulose membranes (Bio-Rad) was performed for one hour at 120 V (constant) in buffer containing methanol and SDS. After checking the efficiency of transfer by staining with Ponceau S, the membranes were blocked with 5% skimmed milk in TTBS (10 mmol Tris/L, 150 mmol NaCl/L, 0.5% Tween 20) overnight at 4°C. AKT and pAKT were detected on the membranes after a two-hour incubation at room temperature with primary antibodies (AKT sc1618, pAKT sc7985-R, Santa Cruz Biotechnology, Santa Cruz, CA) (diluted 1 : 500 in TTBS containing 3% dry skimmed milk). The membranes were then incubated with a secondary specific immunoglobulin G antibody (diluted 1 : 5000 in TTBS containing 3% dry skimmed milk) for two hours at room temperature. Enhanced chemiluminescence (SuperSignal West Pico, Pierce) after incubation with a horseradish peroxidase-conjugated secondary antibody was used for detection by autoradiography. Band intensities were quantified by optical densitometry (Scion Image, Frederick, MD).

### 2.6. Statistical Analysis

The results are presented as the means and SEM for the number of rats (*n*) indicated. Bartlett's test for the homogeneity of variances was initially used to check the fit of the data to the assumptions for parametric analyses of variance. When necessary, data were log-transformed to correct for variance heterogeneity or nonnormality [28]. These data were analyzed by one-way analyses of variance, followed by the LSD test for individual differences between groups. *P* values less than 0.05 were considered to indicate statistically significant differences.

## 3. Results

Absolute food intake was similar between R and C rats and higher in both of these groups compared with LP (*P* < 0.0001). When expressed per gram of body weight, food intake was significantly greater in LP rats than in R rats (*P* < 0.001), while the latter group had significantly higher food intake than C rats (*P* < 0.02). Although R rats had a greater final body weight and epididymal white adipose tissue (EWAT) weight than LP rats (*P* < 0.0001) at the end of the experimental period, their weights were still significantly lower than that of C rats (*P* < 0.05). The R and C rats had similar liver weights, and in both cases these were significantly higher than those of LP rats (*P* < 0.0001). Liver glycogen content was similar in R and LP rats, and both groups exhibited greater liver glycogen concentrations than the C group (*P* < 0.05 and *P* < 0.01, resp.). Serum albumin concentrations do not differ between the R and C rats, and these values were significantly higher than those of LP rats (*P* < 0.0001). The basal serum glucose level was not different among three groups of rats. The basal serum insulin concentration was higher in R rats than in LP rats (*P* < 0.0001) but lower in R rats than in C rats (*P* < 0.01). Serum leptin concentrations did not differ between R and LP rats, and these values were significantly lower than those of C rats (*P* < 0.01) (Table 1).

**Figure 1:**
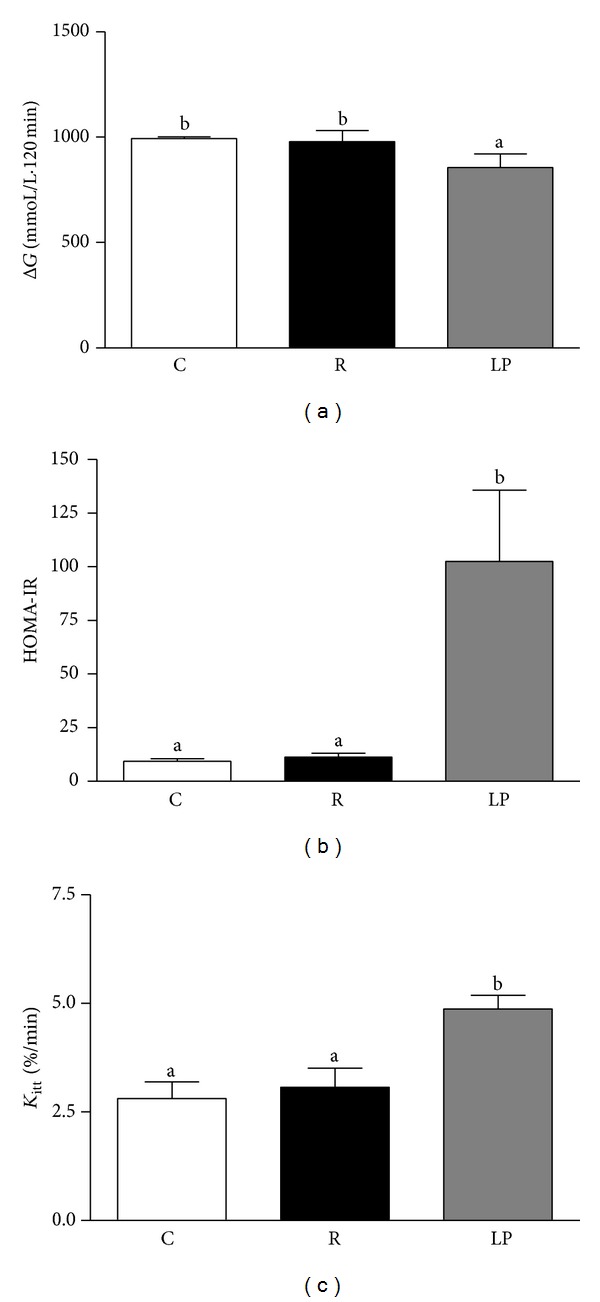
Total areas under the glucose (Δ*G*) curve obtained from the intraperitoneal glucose-tolerance test (a), homeostasis model assessment of insulin resistance (HOMA-IR) index (b), and glucose disappearance rates (*K*_itt_) (c) in control (C), recovered (R), and low protein (LP) rats. The bars represent means ± SEM. Mean values with unlike superscript letters were significantly different (*P* < 0.05, LSD test).

Mean total areas under the Δ*G* in response to an intraperitoneal glucose load were similar in R and C rats and both were higher than LP rats (*P* < 0.03) (Figure 1(a)). Insulin resistance, calculated by the HOMA-IR index, decreased in R and C groups compared to the LP group (*P* < 0.0001) (Figure 1(b)). In contrast, the glucose disappearance rate during the intraperitoneal insulin tolerance test (*K*_itt_) in R rats was similar to that observed in C rats, and both of these values were lower than the disappearance rate in LP rats (*P* < 0.0001) (Figure 1(c)).

**Figure 2:**
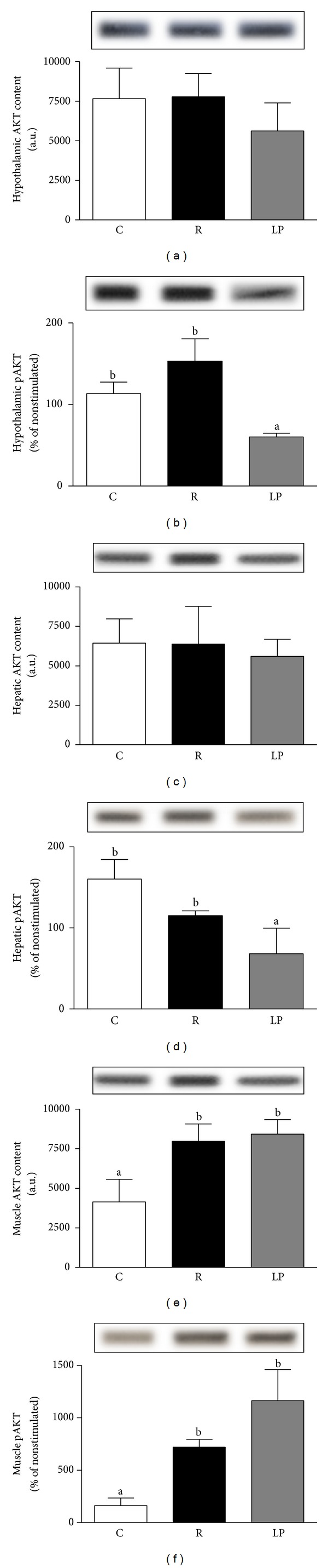
AKT content and AKT phosphorylation in hypothalamus ((a) and (b)), liver ((c) and (d)), and skeletal muscle ((e) and (f)) of control (C), recovered (R), and low protein (LP) rats. AKT phosphorylation data represent the percentage of nonstimulated values. The rats were injected with saline (not shown) or insulin, and 45 s, 90 s, and 15 minutes later, liver, hind-limb skeletal muscle, and hypothalamus, respectively, were excised and homogenized as described in the Materials and Methods section. Data are presented as means ± SEM. Mean values with unlike superscript letters were significantly different (*P* < 0.05, LSD test).

Hypothalamic (Figure 2(a)) and hepatic (Figure 2(c)) AKT content detectable by immunoblotting was not significantly different between the groups. However, after the administration of insulin, the AKT phosphorylation increments in the hypothalamus (Figure 2(b)) and liver (Figure 2(d)) were similar in R and C rats, and the values of these groups were higher than the values of the LP group (*P* < 0.05). In muscle, both the AKT content (Figure 2(e)) and the magnitude of AKT phosphorylation increments (Figure 2(f)) were similar between R and LP groups, and both were higher than the C group (*P* < 0.03 and *P* < 0.01, resp.).

**Figure 3:**
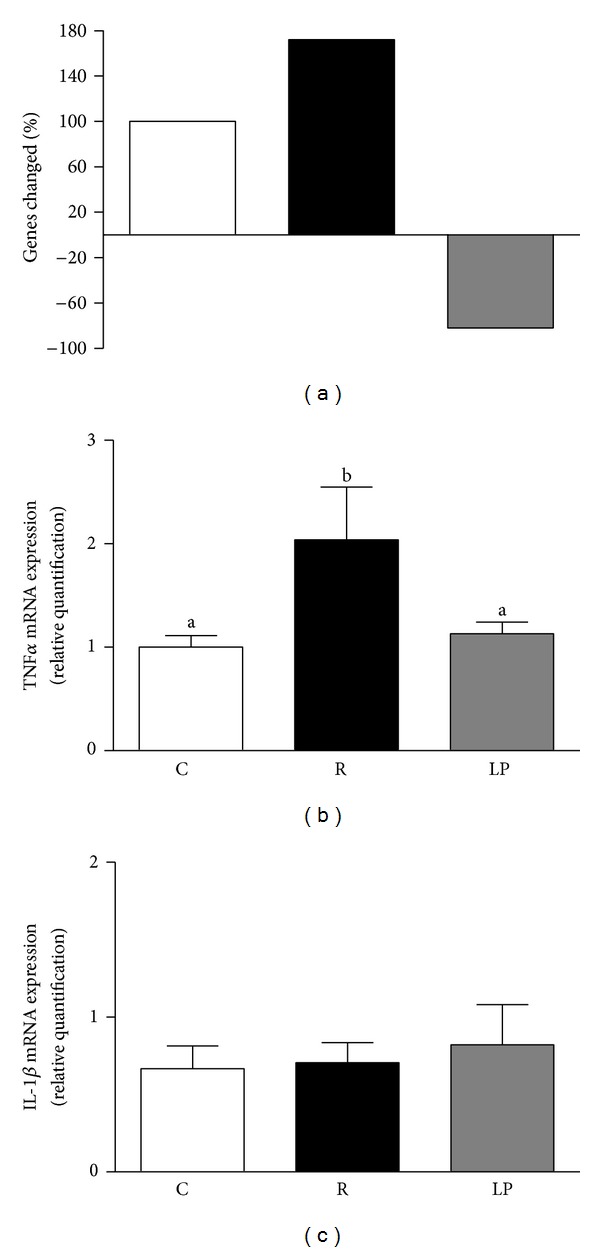
Fold change genes modulated by the experimental treatments evaluated in hypothalamus by PCR array (a). TNFα (b) and IL-1*β* (c) mRNA expression in hypothalamus of control (C), recovered (R), and low protein (LP) rats. Data are presented as means ± SEM. Mean values with unlike superscript letters were significantly different (*P* < 0.05, LSD test).

Of the 84 genes evaluated in the hypothalamus by PCR array (Rat Inflammatory Cytokines Receptors RT² Profiler-SuperArray Bioscience Corp., Frederick, MD, USA), 72 genes (85%) showed a significant change in their level of expression in the R and LP groups compared with the C group. Of the 72 genes changed, 52 (72%) were at least 2.5-fold upregulated in the R group and 59 (82%) were 2.5-fold downregulated in the LP group compared to the C group (Figure 3(a)). Real-time PCR was conducted to validate the findings of the PCR array, and the genes selected for analysis were TNFα and IL*β*. TNF*α* expression was similar between the C and LP groups and smaller in the R group than in the C and LP groups (*P* < 0.5) (Figure 3(b)), whereas IL*β* did not differ among the three groups (Figure 3(c)).

**Figure 4:**
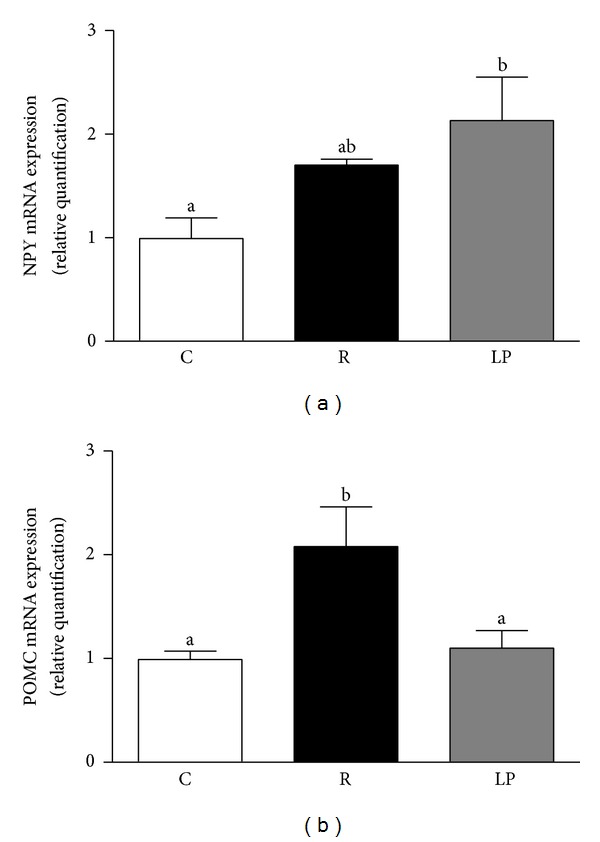
NPY (a) and POMC (b) mRNA expression in hypothalamus of control (C), recovered (R), and low protein (LP) rats. Data are presented as means ± SEM. Mean values with unlike superscript letters were significantly different (*P* < 0.05, LSD test).

Greater NPY mRNA expression was detected in the hypothalamus of LP rats than in those of C rats (*P* < 0.02). NPY mRNA expression was not significantly different between the R rats and either the C or the LP rats (Figure 4(a)). The R group showed higher POMC mRNA expression compared with both the LP and the C groups (*P* < 0.01 and *P* < 0.02, resp.) (Figure 4(b)).

## 4. Discussion

In the present study, we showed that nutritional recovery only attenuated the typical hyperphagia associated with protein malnutrition. Long term feeding regulation is provided by the main circulating hormones leptin and insulin [29], and leptin circulating levels are proportional to total fat mass [30, 31]. In this study, the low visceral fat content correlated with reduced serum leptin levels in recovered and low protein rats. The expected upregulation of hypothalamic NPY mRNA expression and downregulation of hypothalamic POMC mRNA expression in response to low circulating leptin levels [32, 33] was observed in our low protein rats. Although the recovered group also exhibited reduced serum leptin levels, these animals showed hypothalamic POMC mRNA overexpression, most likely due to the higher serum insulin levels in the recovered group compared with the low protein group. Insulin administration increases POMC mRNA expression while reducing NPY expression and protecting against diet-induced obesity [34]. Thus, increased expression of this anorexigenic neuropeptide may have contributed to the attenuation of hyperphagia. However, high hypothalamic TNFα mRNA expression in these animals indicates a hypothalamic inflammatory process that could contribute to incomplete reversion of hyperphagia and development of late obesity.

Inflammation in the hypothalamus leads to insulin resistance, which may play a role in the development of obesity and act as a molecular link between obesity and type 2 diabetes [35–37]. In the present study, nutritional recovery upregulated, whereas low protein diet downregulated, proinflammatory cytokines mRNA expression. Phosphatidylinositol 3-kinase (PI3K)/AKT is an important pathway to control energy homeostasis [38], as it is responsible for the anti-inflammatory response, liver glycogen synthesis, and regulation of insulin-stimulated transport of GLUT4 in muscle [39]. Thus, we initially evaluated hypothalamus AKT signaling and verified that recovered animals did not show impairment in hypothalamic AKT protein expression or phosphorylation. Interestingly, low protein rats that did not exhibit a hypothalamic inflammation signal showed unaltered AKT expression but reduced AKT phosphorylation. As the basal AKT phosphorylation did not differ among groups (data not shown), it is plausible that reduced insulinemia contributed to lower AKT signaling in the hypothalamus of our low protein rats.

Hypothalamic inflammation plays an important role in the development or progression of the hyperglycemic phenotype [40] because glucose homeostasis is controlled by the brain-liver axis [16]. The next step was to evaluate the AKT signaling in the liver, and coincidently, we verified the same pattern in the hypothalamus. The hepatic insulin resistance assessed by AKT phosphorylation level was confirmed by elevated HOMA-IR in low protein rats. The paradoxical association between hepatic insulin resistance and high glycogen levels can be explained by reduced glucose-6-phosphatse activity typically observed in malnourished animals [41]. In low protein rats, the liver insulin resistance was counteracted by the enhanced muscle AKT phosphorylation and expression that resulted in peripheral insulin sensitivity, reflected in the increased *K*_itt_ value. This effect resulted in better glucose tolerance, judging by the low Δ*G* during ipGTT. The persistently high muscle AKT phosphorylation and expression observed in recovered animals may have resulted from incomplete restoration of body weight and serum insulin levels. Lower body weight associated with decreased serum insulin levels apparently has the opposite, compensatory effect [42].

In conclusion, our results are consistent with the hypothesis that protein deprivation produces increased food intake that is partially restored by nutritional recovery. The data also provide direct evidence of maintenance of glucose homeostasis in both low protein and recovered rats. In recovered rats, middle hyperphagia may have resulted from increased anorexigenic neuropeptide expression that counteracted the hypothalamic inflammatory process. In long term protein deprived rats, hyperphagia appears to have been produced by increased orexigenic neuropeptide levels, and glucose homeostasis was maintained, at least in part, at the expense of increased muscle insulin sensitivity.

## Figures and Tables

**Table 1:** Absolute and relative food intake, final body weight, epididymal white adipose tissue (EWAT) weight, liver weight, liver glycogen content, serum albumin, serum glucose, serum insulin, and serum leptin concentrations of control (C), recovered (R), and low protein (LP) rats. Values presented are the mean ± SEM for rats per group. Mean values with unlike superscript letters were significantly different (*P* < 0.05, LSD test).

Parameters	Groups		
	C	R	LP
Food intake (g)	375 ± 19^b^ (15)	351 ± 11^b^ (15)	146 ± 11^a^ (14)
(g/100 g BW)	33.7 ± 0.8^a^ (15)	39.2 ± 1.6^b^ (12)	48.4 ± 2.4^c^ (11)
Body weight (g)	483 ± 11^c^ (15)	444 ± 12^b^ (15)	171 ± 36^a^ (12)
EWAT weight (g)	12.9 ± 0.6^c^ (10)	10.7 ± 0.8^b^ (16)	2.2 ± 0.3^a^ (13)
Liver weight (g)	15.43 ± 0.77^b^ (3)	13.63 ± 1.26^b^ (8)	6.55 ± 0.29^a^ (8)
Liver glycogen content (mg/100 mg)	0.082 ± 0.002^a^ (5)	0.127 ± 0.023^b^ (4)	0.158 ± 0.014^b^ (5)
Serum albumin (g/dL)	3.50 ± 0.04^b^ (7)	3.49 ± 0.03^b^ (7)	3.04 ± 0.02^a^ (7)
Serum glucose (mmol/L)	7.3 ± 0.7 (5)	6.3 ± 0.9 (4)	4.9 ± 1.0 (4)
Serum insulin (ng/mL)	1.7 ± 0.3^c^ (5)	1.0 ± 0.1^b^ (4)	0.11 ± 0.03^a^ (4)
Serum leptin (ng/mL)	4.03 ± 0.05^b^ (4)	3.22 ± 0.28^a^ (4)	2.96 ± 0.16^a^ (4)
